# MRI Findings of Otic and Sinus Barotrauma in Patients with Carbon Monoxide Poisoning during Hyperbaric Oxygen Therapy

**DOI:** 10.1371/journal.pone.0065672

**Published:** 2013-06-12

**Authors:** Ping Wang, Xiao-Ming Zhang, Zhao-Hua Zhai, Pei-Ling Li

**Affiliations:** Sichuan Key Laboratory of Medical Imaging, and Department of Radiology, Affiliated Hospital of North Sichuan Medical College, Shunqing District, Nanchong, Sichuan, China; Harvard Medical School, United States of America

## Abstract

**Background and Purpose:**

To study the MRI findings of otic and sinus barotrauma in patients with carbon monoxide(CO) poisoning during hyperbaric oxygen (HBO) therapy and examine the discrepancies of otic and sinus abnormalities on MRI between barotrauma and acute otitis media with effusion.

**Materials and Methods:**

Eighty patients with CO-poisoning diagnosed with otic and sinus barotrauma after HBO therapy were recruited. Brain MRI was performed to predict delayed encephalopathy. Over the same period, 88 patients with acute otitis media with effusion on MRI served as control. The abnormalities of the middle ear and paranasal sinuses on MRI were noted and were compared between groups. Nine patients with barotrauma were followed up by MRI.

**Results:**

In the barotrauma group, 92.5% of patients had bilateral middle ear abnormalities on MRI, and 60% of patients had both middle ear cavity and mastoid cavity abnormalities on MRI in both ears. Both rates were higher than those in the control group (p = 0.000). In the two groups, most abnormalities on MRI were observed in the mastoid cavity. The rate of sinus abnormalities of barotrauma was 66.3%, which was higher than the 50% in the control group (p = 0.033). In the nine patients with barotrauma followed up by MRI, the otic barotrauma and sinus abnormalities had worsened in 2 patients and 5 patients, respectively.

**Conclusion:**

MRI is able to depict the abnormalities of otic and sinus barotrauma in patients with CO-poisoning during HBO therapy and to differentiate these from acute otitis media with effusion.

## Introduction

HBO therapy has been used widely in clinical practice. It is useful to decrease the incidence of delayed neuropsychologic sequelae in patients with CO-poisoning[1]–[2]. Although patients with CO-poisoning could benefit from the therapy, the side effects, such as barotrauma, should not be overlooked. Barotrauma is defined as tissue damage resulting from the direct effects of pressure[3]–[4]. According to Boyle’s law, a primary change in environmental pressure results in an inverse change in volume. Pressure changes can affect the ears, the sinuses, and the lungs, resulting in injury. Otic barotrauma is one of the most common barotraumas after HBO therapy and is sometimes accompanied by sinus barotraumas [3]–[4]. Under normal conditions, the eustachian tube must open to eliminate the pressure difference between the internal and ambient environments. When the eustachian tube or sinus ostia is blocked or the pressure change exceeds the capacity of its regulation, otic and sinus barotrauma occurs [3]–[8].

Otic barotrauma can cause many discomforts, such as tinnitus, hearing loss, pain and fullness in the sinus and middle ear, and even tympanic membrane perforation. Sinus barotrauma can cause headache, pain and fullness in the sinuses.The diagnosis is based on symptoms and otoscopy or rhinoscopy. The treatments depend on the symptoms and clinical examinations, such as temporary cessation of HBO therapy and the use of decongestants and antibiotics; occasionally, functional middle ear or sinus surgery is needed[2]–[4]. Patients with CO- poisoning and with reduced sensory and cognitive function may have no or slight symptoms of otic and sinus barotrauma after HBO therapy. This situation may lead to a misdiagnosis and/or delay in diagnosis and treatment. Therefore, early diagnosis and differential diagnosis are important to relieve early symptoms and prevent disease progression.

Owing to its high sensitivity to water and fluid on T2-weighted imaging (T2WI) and high sensitivity to hemorrhage on T2WI, magnetic resonance imaging (MRI) has advantages for observing the abnormalities of the middle ear and paranasal sinuses, such as mucosal edema, fluid exudation and subperiosteal hemorrhage. Some studies have reported the MRI findings of temporal bone abnormalities [9]–[12]. On MR images, air and bone are black, and it is easy to distinguish air or bone from mucosal edema or hemorrhage. MRI can also be used to investigate the abnormalities of sinuses. Mucosal thickening in paranasal sinuses exceeding 3 mm is considered abnormal[13]–[16]. Segev et al [17] reported that frontal sinus barotrauma of a flight passenger showed hyperintensity on both T1WI and T2WI sequences, which was consistent with subacute submucosal hemorrhage. MRI is available to depict the early and subtle mucosal changes of the middle ear and paranasal sinuses, and it has the potential to observe otic and sinus barotrauma.

To our knowledge, no studies have investigated the MRI abnormalities of otic and sinus barotrauma. We conducted this study to retrospectively investigate otic and sinus barotrauma by MRI. We hypothesized the following: (1) MRI can depict otic and sinus barotrauma, (2) MRI can differentiate otic and sinus barotrauma from acute otitis media and sinusitis, and (3) MRI can be used to follow the progress of otic and sinus barotrauma.

## Materials and Methods

### Ethics Statement

The study was approved by the Institutional Review Board and the Ethics Committee of Affiliated Hospital of North Sichuan Medical College. Patient informed consent was not obtained.This is because that it’s difficult to get informed consent from all patients involved in our study due to the retrospective nature of this study,and also because that the data were analyzed anonymously in accordance with IRB guidelines. The Ethics Committee of Affiliated Hospital of North Sichuan Medical College waived the need for written informed consent from the participants.The study complies with the ethical principles of the Helsinki Declaration of 1964,revised by the World Medical Organization in Edinburgh in 2000.

### Patient Study

From January 2010 to March 2012, a computer search identified 90 patients with CO-poisoning who underwent head MRI at our institution to predict delayed encephalopathy.

The recruitment criteria for patients with otic and sinus barotrauma in this study were as follows: (1) new onset of otic and sinus symptoms, such as tinnitus, hearing loss and pain or fullness in the ear and paranasal sinus after HBO therapy; (2) clinical examination of the ear, nose, and throat; (3) otoscopy or rhinoscopy during HBO therapy; (4) brain MRI performed after 3 sessions of HBO therapy [18].

The exclusion criteria were (1) artifacts (e.g., motion artifacts and denture artifacts); (2) any one of the middle ear cavity, mastoid cavity, and paranasal sinuses was not covered by MRI; (3) other diseases inducing otic and sinus abnormalities.

During the same period, 94 patients with acute otitis media who underwent MRI for suspicion of intracranial pathology served as the control group. The diagnosis standard of acute otitis media with effusion included (1) new onset of otic and sinus symptoms, such as tinnitus, hearing loss and pain or fullness in the ear; (2) clinical examination of the ear; (3) otoscopy or rhinoscopy; (4) brain MR examinations performed in the acute phase of symptoms [19]–[20].

Ultimately, the barotrauma group consisted of 80 consecutive patients with barotrauma, and the control group consisted of 88 patients with acute otitis media with effusion. Nine cases of barotrauma were followed up successfully.

Ten patients with barotrauma were excluded, including 4 patients with motion artifacts, 2 patients with denture artifacts, and 4 patients without coverage of all paranasal sinuses on MRI. Six patients with acute otitis media were excluded, including 5 patients with denture artifacts and 1 patient without coverage of all paranasal sinuses on MRI. A monoplace-type chamber (YYC08A-1, Hangzhou xinying) was used for HBO therapy. The patient entered the chamber and received 100% O_2_ under a pressure of 2 absolute atmospheres (ATA) for 60 min. The increase and decrease of pressure were carried out within a 10 to 15 min period. According to their condition, patients underwent several such sessions.

### MRI Techniques

All MR examinations were performed with a 1.5-T system and a phased-array coil (Signa, GE Medical Systems, Milwaukee, WI). The sequences observed included axial fast-recovery fast spin-echo (FRFSE) T2-weighted, axial spoiled gradient-echo (SPGR) T1-weighted and axial fluid-attenuated inversion recovery (FLAIR) T2-weighted. The parameters of axial FRFSE T2-weighted MR images were as follows: repetition time (TR), 4000 ms; echo time (TE),100 ms; section thickness, 7 mm; intersection gap, 1 mm; matrix size, 320×192; and field of view (FOV), 24× 24 cm. The parameters of axial SPGR T1-weighted MR images were as follows: TR/TE, 135/1.8 ms; section thickness, 7 mm; intersection gap, 1 mm; matrix size, 256×192; and FOV, 24× 24 cm. The parameters of axial FLAIR T2-weighted MR images were as follows: TR/TE, 8000/108 ms; section thickness, 7 mm; intersection gap, 1 mm; matrix size, 512; and FOV, 24× 24 cm.

### MRI Image Review

The original MRI data were loaded onto a computer workstation (GE Advantage Workstation version 4.1; Sun Microsystems, Palo Alto, CA) for review. Two observers (with 4 and 6 years of experience in interpreting brain MRI examinations, respectively) who were blinded to the clinical outcomes reviewed the MR images. Any discrepancies between the two readers were settled by consensus.

On MR images, air and bone are black, and the signal intensity of normal mucosa in the mastoid cavity and middle ear cavity is invalid. When the middle ear shows slight hypointensity, isointensity or slight hyperintensity on T1WI or hyperintensity on T2WI, it is considered abnormal [11]. The abnormalities of otic barotrauma on MRI can be divided into 3 types according to their sectional anatomic site: (1) middle ear cavity and mastoid cavity; (2) middle ear cavity alone; (3) mastoid cavity alone[9]–[11], [21]–[23]. The severity of otic barotrauma on MRI was graded according to the number of anatomic sites involved. Each anatomic site involving two ears was recorded as 1 point. Thus, one site involving two ears was recorded as 1 point, two sites involving two ears was recorded as 2 points, three sites involving two ears was recorded as 3 points, and four sites involving two ears was recorded as 4 points. Because there are cyclical changes in the nasal cavity, turbinate and sphenoid sinus, the middle ear and paranasal sinus abnormalities on MRI have some discrepancies. In MRI, the nasal mucosal thickening and signal intensities alternate from one side to the other several times every 24 h [24]. During nasal cyclical changes, mucosal thickening up to 2 mm can be normal, but mucosal thickening exceeding 3 mm is considered abnormal [24]. The MRI findings of sinusitis on T1WI and T2WI included (1) a mucosal lining exceeding an estimated thickness of 3 mm, (2) a near-total or total sinus opacification, and (3) an air-fluid level[14]–[16], [24]–[25]. When one of abnormalities above was observed in at least one paranasal sinus, the sinus was defined as abnormal[14], [24]–[25].

### Statistical Analysis

All of the data derived from the MR images were averaged between the two observers. Any discrepancies in noncontinuous data were discussed by the two observers until a consensus was reached.

The chi-square test was used to evaluate the correlation of MRI findings in the middle ear cavity and/or mastoid cavity and/or paranasal sinuses between the barotrauma group and the control group. Data analysis was performed using the Statistical Package for the Social Sciences (SPSS) for Windows version 13.0 (SPSS, Inc, Chicago, IL). Statistical significance was indicated by a p-value of less than 0.05.

## Results

### 1. Patient Sample

In the 80 patients with barotrauma, there were 30 men and 50 women with an average age of 47.2±16.4 years (range 5–78 years). In the control group, the 88 patients included 48 men and 40 women with an average age of 47.3±16.6 years (range 3–83 years). No significant difference was found in age between the two groups.

In the barotrauma group, the therapy depended on the clinical symptoms and otoscopy or rhinoscopy findings. For patients with mild symptoms and no abnormalities of the middle ear or sinus on otoscopy or rhinoscopy, follow-up was needed. For patients with moderate to severe symptoms and abnormalities of the middle ear and sinus on otoscopy or rhinoscopy, compound ofloxacin nasal drops were used, and the patients were asked to do Valsalva maneuver. When the tympanic membrane and mucosa had severe congestion edema, glucocorticoids were administrated to reduce the edema. If symptoms persisted, functional middle ear or sinus surgery was performed. The symptoms or abnormalities of the middle ear on otoscopy were relieved in 70 patients and were not relieved in 10 patients. In 2 patients with long-term follow-up, the symptoms or abnormalities of the middle ear on otoscopy became worse. In 40 of 55 patients with symptoms or abnormalities of the sinus on rhinoscopy, the symptoms or abnormalities were relieved, but they were not relieved in 15 patients. In 5 patients with long-term follow-up, the symptoms or abnormalities of the sinus on rhinoscopy became worse.

### 2. MRI Findings of Barotrauma

**Figure 1:**
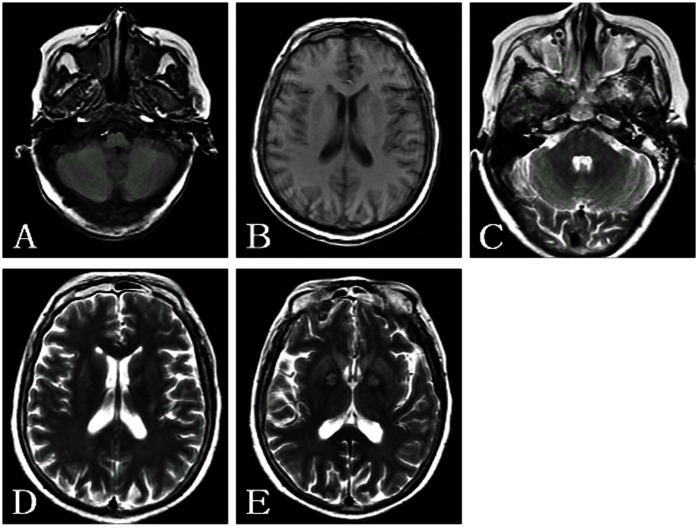
A 61-year-old woman with barotrauma of the left middle ear, bilateral maxillary sinuses and bilateral frontal sinuses. A and C : The left middle ear cavity and mastoid cavity show isointensity or slight hyperintensity on T1WI and hyperintensity on T2WI. A–E: The bilateral maxillary sinuses and frontal sinuses show hypointensity or isointensity on T1WI and hyperintensity on T2WI. E: The symmetric hyperintensity in the bilateral globus pallidus can be observed on T2WI.

**Figure 2:**
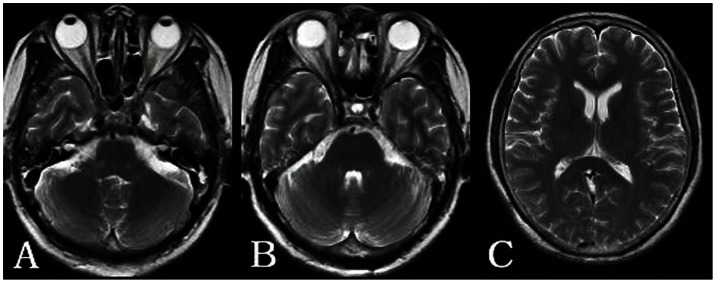
A 60-year-old woman with barotrauma of the bilateral middle ear and ethmoidal sinuses. A: The bilateral mastoid cavities show hyperintensity on T2WI. A–B: The bilateral ethmoidal sinuses show hyperintensity on T2WI. C: No abnormality can be observed in the brain on T2WI.

**Figure 3:**
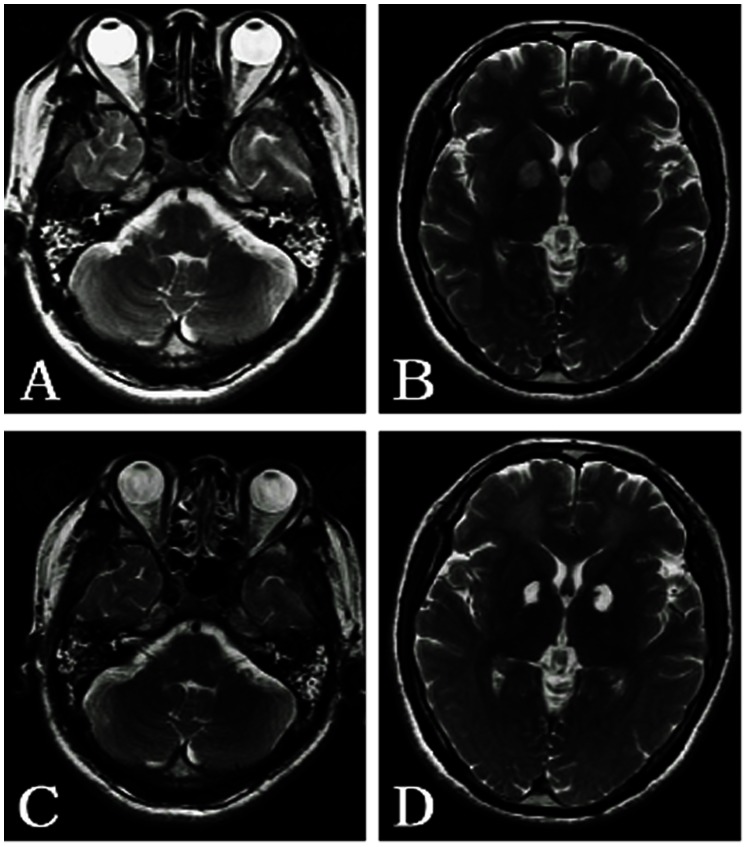
A 38-year-old man with barotrauma of the bilateral middle ears and right ethmoidal sinus. A: The bilateral mastoid cavities, left middle ear cavity and right ethmoidal sinus show hyperintensity on T2WI. B: The bilateral globus pallidus shows symmetric hyperintensity on T2WI. C: Three months later, the same sites above show hyperintensity on T2WI, but the mucosa in right ethmoidal sinus is thicker than in the initial scan. D: The symmetric hyperintensity can be observed in the bilateral globus pallidus and in the bilateral white matter on T2WI.

**Figure 4:**
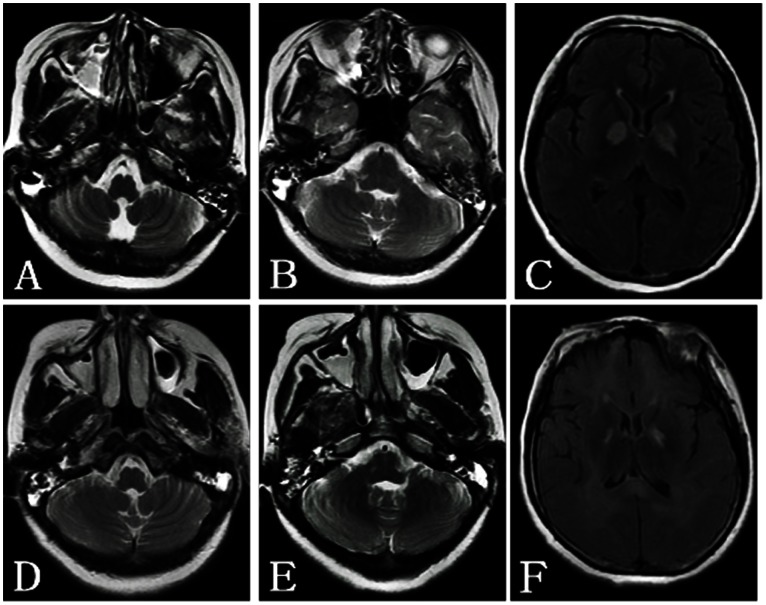
A 41-year-old woman with barotrauma of the bilateral middle ears and right maxillary sinus. A–B: The bilateral mastoid cavities, left middle ear cavity and right maxillary sinus show hyperintensity on T2WI. C: The bilateral globus pallidus shows symmetric hyperintensity on T2WI FLAIR. D-E: Two months later, the left maxillary sinuses, and the sphenoid sinus besides the sites above show hyperintensity on T2WI. F: The symmetric hyperintensity can be observed in the bilateral globus pallidus and the bilateral white matter on T2WI FLAIR.

All 80 patients with barotrauma had at least one abnormality of two middle ears on MRI. The rate of bilateral abnormalities of the middle ear on MRI was 92.5% (74/80), and the rate of unilateral abnormalities of the middle ear on MRI was 7.5% (6/80). According to the MR severity scores, 3.75% (3/80), 30% (24/80), 6.25% (5/80) and 60.0% (48/80) of patients were, respectively, recorded as 1 point, 2 points, 3 points and 4 points (Fig. 1, 2, 3, 4).

Among the 160 ears of the 80 patients with barotrauma, 97.5% (156/160) of the middle ears had mastoid cavity abnormalities, and 68.8% (110/160) of the middle ears had middle ear cavity abnormalities on MRI.

In the 80 patients with barotrauma, 66.3% (53/80) of patients had abnormalities in paranasal sinuses (Fig. 1, 2, 3, 4). Additionally, 43.8% (35/80) of patients had maxillary sinus abnormalities, which was the most commonly involved site, and 42.5% (34/80) of patients had ethmoid sinus abnormalities, which was the second-most common site.

**Figure 5:**
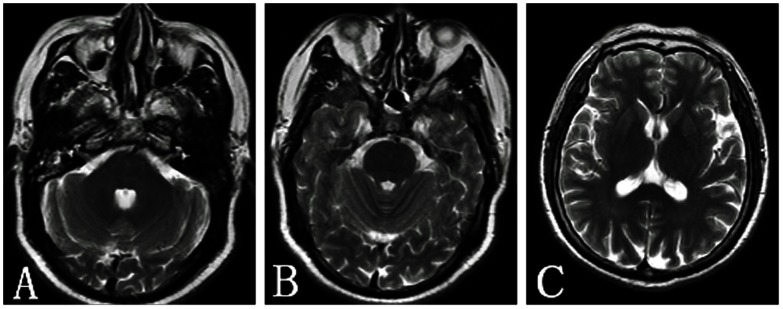
A 47-year-old man with acute otitis media with effusion of MRI abnormalities in the right middle ear, bilateral maxillary sinuses and sphenoid sinus. A: The right mastoid cavity and middle ear cavity show hyperintensity on T2WI. A–B: The bilateral maxillary sinuses and sphenoid sinus show hyperintensity on T2WI. C: No abnormality can be observed in the brain on T2WI.

All 88 patients of the control group had at least one abnormality of two middle ears on MRI. The rate of bilateral abnormalities of the middle ear in the control group on MRI was 50% (44/88), and the rate of unilateral abnormalities of the middle ear on MRI was 50% (44/88). According to the MR severity scores, 14.8% (13/88), 58.0% (51/88), 13.6% (12/88) and 13.6% (12/88) of patients were, respectively, recorded as 1 point, 2 points, 3 points and 4 points (Fig. 5).

In the two ears, 80.1% (141/176) of middle ears showed mastoid cavity abnormalities, and 35.8% (63/176) of middle ears had middle ear cavity abnormalities on MRI.

In the 88 patients of the control group, 50% (44/88) of patients had paranasal sinus abnormalities on MRI (Fig. 5), and 39.8% (35/88) of patients had maxillary sinus abnormalities, which was the most commonly involved site, while 30.7% (27/88) of patients had ethmoid sinus abnormalities on MRI, which was the second-most common site.

### 3. The MRI Findings of Barotraumas Compared with the Control Group

The rate of bilateral middle ear abnormalities on MRI was 92.5% in the barotrauma group, which was higher than the 50% in the control group (p = 0.000) (Table 1). The rate of unilateral middle ear abnormalities on MRI was 7.5% in the barotrauma group, which was lower than the 50% in the control group (p = 0.000). The rate of abnormalities with a MR severity score of 4 points (i.e., bilateral simultaneous abnormalities of both the middle ear cavity and mastoid cavity) in the barotrauma group was 60%, which was higher than the 13.6% in the control group (p = 0.000).

**Table 1:** The severity of otic barotrauma on MRI.

MR score	1 point		2 points			3 points	4 points
	Right ear	Left ear	Right ear	Left ear	Bilateral ears		
Barotrauma group (n = 80)	3	0	1	2	21	5	48*
Control group (n = 88)	11	2	12	19	20	12	12

Note:* Chi-square, p<0.05 compared to control group.

The rate of mastoid cavity abnormalities in both ears on MRI was 97.5% in the barotrauma group, which was higher than the 80.1% in the control group (p = 0.000). The rate of middle ear cavity abnormalities in both ears on MRI was 68.8% in the barotrauma group, which was higher the 35.8% in the control group (p = 0.000). In both groups, the rate of abnormalities in the mastoid cavities of both ears was higher than that of abnormalities in the middle ear cavity on MRI (P = 0.000).

The rate of paranasal sinus abnormalities was 66.3% in the barotrauma group and 50% in the control group (p = 0.033) (Table 2). There was also a significant difference in the rate of sphenoid sinus abnormalities between the two groups (25.0% vs 11.4%, p = 0.021).

**Table 2:** The paranasal sinus abnormalities on MRI.

Abnormality	Barotrauma group (n = 80)	Control group (n = 88)
Maxillary sinuses	35 (43.8%)	35 (39.8%)
Ethmoidal sinuses	34 (42.5%)	27 (30.7%)
Sphenoid sinus	20 (25%)*	10 (11.4%)
Frontal sinuses	8 (10%)	6 (6.8%)

Note: *Chi-square, p<0.05 compared to the acute otitis media group.

### 4. MRI Follow-up for Patients with Barotrauma

In 9 patients with otic barotrauma, 5, 1 and 3 patients received MR severity scores of 4 points, 3 points and 2 points, respectively, in the initial MRI scan. In the follow-up MRI, 7, 1, and 1 patient received 4 points, 3 points, and 2 points, respectively. Two patients with coma during hyperbaric oxygen therapy had more sites of abnormalities on MRI in the follow-up scan compared to baseline, with the MR severity scores rising from 2 points to 4 points, and they also had exacerbations of the symptoms of pain or fullness in the middle ear. These findings are consistent with the brain MRI findings (Fig. 3, 4). The other 7 patients had no more sites of abnormalities on MRI and no exacerbations of symptoms of middle ear pain or fullness. Their state of consciousness during HBO therapy was sober. The brain abnormalities on MRI included no exacerbations. Patient consciousness during HBO therapy had some effects on the prognosis of otic barotrauma. The patients who lost consciousness during HBO therapy were more likely to experience exacerbations of otic barotrauma. These data indicate that MR follow-up is needed for patients with otic barotrauma, especially patients who lose consciousness during HBO therapy. Some early treatments are also needed.

Of the 9 patients with barotrauma who were followed up, 5 patients had sinus abnormalities in the initial MRI scan. In these 5 patients, 4, 3, 2, and 1 patients had abnormalities of the maxillary sinus, ethmoid sinus, sphenoid sinus, and frontal sinus on the initial MRI, respectively (Table 3). In the follow-up scan, the same 5 patients had sinus abnormalities, but the number of sinus abnormalities on MRI increased. There were 5, 5, 2, and 1 patients with abnormalities of the maxillary sinus, ethmoid sinus, sphenoid sinus, and frontal sinus in the follow-up MRI scan, respectively. All reported exacerbations of the symptoms of pain or fullness in the respective sinuses, and all had more sites of sinus abnormalities than before (Fig. 3, 4). The other 4 patients, without sinus abnormalities in the initial MRI scan, still had no sinus abnormalities in the follow-up MRI, nor any exacerbations of their symptoms of pain or fullness in the sinus. Overall, our findings suggest that the sinus abnormalities of barotrauma on MRI will become aggravated over time, and timely treatment is needed for patients with sinus abnormalities on MRI.

**Table 3:** Results of the follow-up MRI for sinus barotrauma(n = 9).

	Maxillary sinus abnormal	Ethmoid sinus abnormal	Sphenoid sinus abnormal	Frontal sinus abnormal
Initial scan	4	3	2	1
Follow-up	5	5	2	1

## Discussion

Barotrauma is pressure-induced injury. MRI can be useful to evaluate effusions in the middle ear and sinus disease [14], [21]–[25]. Our data reveal that in the group of patients who suffered from barotrauma, most of the abnormalities were found in the bilateral middle ear, and in the bilateral abnormalities, simultaneous abnormalities of the middle ear cavity and mastoid cavity are the most common. The rate of middle ear and sinus abnormalities of barotrauma was significantly higher than in the control group, including simultaneous abnormalities of the bilateral middle ear cavity and mastoid cavity, abnormalities of either middle ear cavity or mastoid cavity of the two ears, and abnormalities of the sphenoid sinus. MR imaging is useful to distinguish otic or sinus barotrauma from acute otitis media with effusion. In both groups of this study, the abnormalities of the middle ear were most commonly and first observed in the mastoid cavity. Our results indicate that bilateral middle ear abnormalities on MRI during HBO, especially both middle ear cavities and mastoid cavities, with no pre-existing history of otologic disease,could be considered barotrauma. When mastoid cavity abnormalities occur on MRI, steps should be taken to prevent the involvement of the middle ear cavity, or else delayed healing and disease progression are likely.

We found that the middle ear cavity and mastoid cavity abnormalities in otic barotrauma can be observed on MRI, and the mastoid cavity is the most frequently involved. One reason for this may be as follows: The human mastoid as well as the eustachian tube is capable of active counter-regulation of the middle ear pressure. The mastoid air cell system serves as the middle ear gas reserve and is the buffer of middle ear pressure owing to the higher surface area/volume ratio of the former, which is related to the continuous regulation of smaller pressures, whereas the eustachian tube is responsible for the intermittent regulation of higher pressures by communicating with the atmosphere and balancing internal air pressure with the atmosphere [26]–[31]. The opening of the eustachian tube might be affected by upper respiratory tract infections, a history of otitis media, or relative negative middle ear pressure while undergoing HBO treatments [32]. When the eustachian tube is dysfunctional, the mastoid cell system becomes the main regulator of ear pressure. It regulates the pressure through mucosal thickness and changes in the diffusion of gas and fluid [26], [27]. This may contribute to the hyperintensity of the mucosal thickening and the fluid exudation in the mastoid cavity. Because the mastoid cell system is the gas reserve and buffer system of the middle ear, enabling gradual and slow pressure changes [27], it may be affected more strongly and earlier than other areas (e.g., tympanic membrane retraction, the rupture of blood vessels, the damage of ear mucosa, fluid exudation into the middle ear space, and even tympanic membrane perforation [3], [4]). Consequently, the mastoid cavity is the most frequently involved site according to MRI.

Patients with history of sinusitis, otitis media or an upper respiratory infections may have a higher frequency of sinus barotrauma [6], [7], [33]. This could be because the sinus cavities communicate with the nasal cavities through ostia and through a long and tortuous duct in the sinuses. When the sinus ostia is blocked, the pressure is disequilibrated between the sinuses and nasal cavity. The increasing ambient pressure is transmitted through the sinus wall, leading to vascular congestion and edema of the sinus mucosa. When the elastic limit of the mucosa is exceeded, hemorrhage will occur [34]. In our study, the maxillary sinuses were the most commonly involved sites, which is in agreement with Fagan’s study [6], and the ethmoid sinuses were the second-most commonly involved sinuses.

In our study the rate of middle ear and sinus abnormalities of barotrauma was significantly higher than in the control group. This may have been due to the effect of the negative pressure in middle ears and sinuses [2]–[4]. The bilateral middle ear abnormalities were more frequently observed in the patients with barotrauma than the control group. This is because the chance of a patient being affected by HBO treatments is the same in each ear, which is higher than that of the control group. In acute otitis media with effusion, tubal function, the degree of mastoid pneumatization, nasopharyngeal colonization with pathogenic bacteria and viruses, an infectious pathway that ascends along the eustachian tube, the immune status of the host, allergic and environmental factors, and genetic predisposition all contribute to the disease status [35]. The possibility of abnormalities is equal in each ear, but the rate of bilateral ear involvement is small. Owing to the negative pressure in the sinuses induced by HBO treatments [4], the rate of abnormalities in the barotrauma group was higher than that of the control group. Abnormalities of the sphenoid sinus are rare, representing 1–2.7% of those with paranasal sinus disease or paranasal sinusitis [36]. Because of the relative negative pressure in the sphenoid sinus [7], the chance of sphenoid sinus involvement in patients with barotrauma is higher than in control patients.

In both groups, the mastoid cavity was the most frequently involved site on MRI. The reason may be the gas reserve and buffer effect of the mastoid cell system[29]–[31]. The opening of the eustachian tube is mainly affected by an upper respiratory infections in patients with acute otitis media with effusion while by relative negative middle ear pressure in patients with barotrauma [4], [19]. This may induce dysfunction of eustachian tube of gas balance, and the main regulator of the middle ear pressure is the mastoid cell system[29]–[31]. It regulates the pressure through mucosal thickness and changes in the diffusion of gas and fluid [26], [27]. So the mastoid cavity was the most site observed abnormality on MRI in both groups.

Barotrauma is a pressure-induced injury, and can induce exudation of aseptic [7]. Uzun C [7] reports the exudation of aseptic can be absorbed by body itself. The management of barotrauma is usually based on the patient’s clinical symptoms [32]. In our study, the patients who lost consciousness had exacerbations of otic barotrauma, some of which were consistent with the exacerbations of brain MRI findings. This may be explained as follows: Patients undergoing HBO therapy need to perform the movements of swallowing, opening and closing the mouth, and pinching the nose to maintain the balance of pressure in the middle ear [4]. The opening of the eustachian tube is affected more strongly when patients lose consciousness, so their ability to spontaneously balance pressure decreases during the period of HBO therapy [4]. In the period between HBO therapy sessions, patients perform the Valsalva maneuver less frequently due to coma, and they do not inform the doctors of their middle ear symptoms early enough, so the otic barotrauma recovery rate is low [4]. This may induce the exacerbations of otic barotrauma and middle ear abnormalities observed on MRI. The condition of consciousness may be a risk factor for cognitive sequelae, which may affects the prognosis of CO-poisoning [37]. When patients are in a coma, the incidence of delayed encephalopathy increases, and so does the rate of brain abnormalities on MRI [37]. The exacerbations of otic barotrauma on MRI in our patient population are consistent with the exacerbations of brain MRI findings.

In our study, the middle ear and sinus abnormalities on MRI had become worse upon follow-up in some cases, which is consistent with the findings of Campell et al [34], [36], [38]. They suggested that repetitive barotrauma could lead to permanent changes in the paranasal sinuses. In our study, the patients with abnormalities in the initial MR scan all had more serious MR findings in the follow-up scan. The possible causes of such exacerbations are the following: (1) The process of barotrauma is initiated with avulsion of mucosa from the periosteum, and then substantial mucosal thickening increases the potential for ostial narrowing and also induces negative pressure in the middle ears and sinuses [35], [38]. (2) We defined abnormal sinus MRI findings as a mucosal lining exceeding an estimated thickness of 3 mm, a near-total or total sinus opacification, and an air-fluid level. These should be considered moderate or severe abnormalities, and they may infrequently be self-limited. (3) The treatment of barotrauma depends on the symptoms or the otoscopy or rhinoscopy findings, and only the patients with severe symptoms or otoscopy or rhinoscopy findings are referred to an otolaryngologist [39]. (4) Patients did not undergo a MRI examination or otoscopy or rhinoscopy before HBO therapy, so we did not exclude the possibility of asymptomatic sinusitis before MRI. Our results indicate that otic and sinus barotrauma sometimes is not self-limited, so follow-up MRI and appropriate management are necessary. When patients have sinus abnormalities in the initial MR scan, the sinuses should be treated to prevent the further development of sinus barotrauma.

A limitation of this study is that the included patients did not undergo MRI, otoscopy, or rhinoscopy before HBO therapy. Because CO-poisoning can cause potentially permanent neurologic deterioration, HBO therapy is performed as soon as possible, before MRI [1]. In our study, patients with history of otic and sinus symptoms before HBO therapy were excluded, so the above factors should have had a small effect on our results. Another limitation is that the number of patients followed up was small. In the future, a large group of patients with barotrauma and who are followed up may be needed to study the correlation of MRI findings with the prognosis of barotrauma.

### Conclusion

Most of the otic abnormalities in the barotrauma group were bilateral and involved both the mastoid cavity and the middle ear cavity. The rate of otic and sinus abnormalities were higher than in the control group. Being aware of these features can be valuable when distinguishing otic and sinus barotrauma from acute otitis media with effusion. Follow-up MRI and appropriate management for the patients with otic and sinus barotrauma are necessary to prevent further development of the lesion.
